# Supplementary material for: Modeling the Angle-Specific Isokinetic Hamstring to Quadriceps Ratio Using Multilevel Generalized Additive Models
Source: Medicina (Kaunas). 2019 Jul 26;55(8):411. doi: 10.3390/medicina55080411 (PMC6722624; doi:10.3390/medicina55080411)
Supplement: Supplementary file 1 [file medicina-55-00411-s001.pdf]

SUPPLEMENTARY MATERIAL FOR THE PAPER "MODELING ANGLE-SPECIFIC ISOKINETIC HAMSTRING TO QUADRICEPS RATIO USING MULTILEVEL GENERALIZED ADDITIVE MODELS"

R code for the models of isokinetic strength curves using generalized additive multilevel models

```
#concentric knee extension

ke1<-brm(bf(kn_ext_cc_Nm ~ s(angle_ext_ref0) + (angle_ext_ref0|id)),
      data = ke,
      family = gaussian(),
      chains = 4,
      control = list(adapt_delta = 0.95,max_treedepth = 15))


summary(ke1)
Family: gaussian
  Links: mu = identity; sigma = identity
Formula: kn_ext_cc_Nm ~ s(angle_ext_ref0) + (angle_ext_ref0 | id)
   Data: ke (Number of observations: 2319)
Samples: 2 chains, each with iter = 2000; warmup = 1000; thin = 1;
         total post-warmup samples = 2000

Smooth Terms:
                       Estimate Est.Error l-95% CI u-95% CI Eff.Sample Rhat
sds(sangle_ext_ref0_1)   162.54     39.70   103.59   258.84        500 1.01

Group-Level Effects:
~id (Number of levels: 30)
                              Estimate Est.Error l-95% CI u-95% CI Eff.Sample Rhat
sd(Intercept)                    39.25      5.58    30.22    52.20        441 1.01
sd(angle_ext_ref0)                0.81      0.11     0.63     1.06        450 1.00
cor(Intercept,angle_ext_ref0)    -0.35      0.16    -0.64    -0.02        403 1.00

Population-Level Effects:
                 Estimate Est.Error l-95% CI u-95% CI Eff.Sample Rhat
Intercept          138.24      7.81   123.40   153.25        296 1.01
sangle_ext_ref0_1  -83.71      9.22  -101.85   -66.16       1025 1.00

Family Specific Parameters:
      Estimate Est.Error l-95% CI u-95% CI Eff.Sample Rhat
sigma    10.73      0.16    10.43    11.04       1693 1.00

Samples were drawn using sampling(NUTS). For each parameter, Eff.Sample
is a crude measure of effective sample size, and Rhat is the potential
scale reduction factor on split chains (at convergence, Rhat = 1).
```

```
#eccentric knee extension


ke2<-brm(bf(kn_ext_ecc_Nm ~ s(angle_ext_ref0) + (angle_ext_ref0|id)),
      data = ke,
      family = gaussian(),
      chains = 4,
      control = list(adapt_delta = 0.95,max_treedepth = 15))


summary(ke2)

Family: gaussian
  Links: mu = identity; sigma = identity
Formula: kn_ext_ecc_Nm ~ s(angle_ext_ref0) + (angle_ext_ref0 | id)
   Data: ke (Number of observations: 2310)
Samples: 2 chains, each with iter = 4000; warmup = 1000; thin = 1;
         total post-warmup samples = 6000

Smooth Terms:
                       Estimate Est.Error l-95% CI u-95% CI Eff.Sample Rhat
sds(sangle_ext_ref0_1)    45.05     14.37    24.56    79.68       2287 1.00

Group-Level Effects:
~id (Number of levels: 30)
                              Estimate Est.Error l-95% CI u-95% CI Eff.Sample Rhat
sd(Intercept)                    57.21      7.66    44.08    74.27       1397 1.00
sd(angle_ext_ref0)                0.66      0.09     0.51     0.87       1562 1.00
cor(Intercept,angle_ext_ref0)    -0.76      0.08    -0.88    -0.57       2095 1.00

Population-Level Effects:
                  Estimate Est.Error l-95% CI u-95% CI Eff.Sample Rhat
Intercept           134.01      7.27   119.69   148.32       1208 1.00
sangle_ext_ref0_1   -14.01      6.50   -26.48    -1.21       3017 1.00

Family Specific Parameters:
      Estimate Est.Error l-95% CI u-95% CI Eff.Sample Rhat
sigma    12.25      0.18    11.90    12.62       6000 1.00

Samples were drawn using sampling(NUTS). For each parameter, Eff.Sample
is a crude measure of effective sample size, and Rhat is the potential
scale reduction factor on split chains (at convergence, Rhat = 1).
```

```
#hamstrings by quadriceps at each angular position as dependent variable"

ke3<-brm(bf(H_Q~ s(angle_ext_ref0) + (angle_ext_ref0|id)),
      data = ke,
      family = gaussian(),
      chains = 4,
      control = list(adapt_delta = 0.99,max_treedepth = 15))

summary(ke3)
Family: gaussian
  Links: mu = identity; sigma = identity
Formula: H_Q ~ s(angle_ext_ref0) + (angle_ext_ref0 | id)
   Data: ke (Number of observations: 2319)
Samples: 2 chains, each with iter = 4000; warmup = 1000; thin = 1;
         total post-warmup samples = 6000

Smooth Terms:
                       Estimate Est.Error l-95% CI u-95% CI Eff.Sample Rhat
sds(sangle_ext_ref0_1)     2.76      0.94     1.53     5.08       1387 1.00

Group-Level Effects:
~id (Number of levels: 30)
                              Estimate Est.Error l-95% CI u-95% CI Eff.Sample Rhat
sd(Intercept)                     0.94      0.13     0.73     1.24        654 1.00
sd(angle_ext_ref0)                0.02      0.00     0.01     0.02        682 1.00
cor(Intercept,angle_ext_ref0)    -0.98      0.01    -0.99    -0.96        809 1.00

Population-Level Effects:
                  Estimate Est.Error l-95% CI u-95% CI Eff.Sample Rhat
Intercept             1.23      0.06     1.12     1.34        473 1.00
sangle_ext_ref0_1    -2.40      0.29    -2.99    -1.85       1859 1.00

Family Specific Parameters:
      Estimate Est.Error l-95% CI u-95% CI Eff.Sample Rhat
sigma     0.39      0.01     0.38     0.40       6000 1.00

Samples were drawn using sampling(NUTS). For each parameter, Eff.Sample
is a crude measure of effective sample size, and Rhat is the potential
scale reduction factor on split chains (at convergence, Rhat = 1).
```

```
#knee flexion#


kf1<-brm(bf(kn_flex_cc_Nm ~ s(angle_ext_ref0) + (angle_ext_ref0|id)),
      data = kf,
      family = gaussian(),
      chains = 4,
      control = list(adapt_delta = 0.95,max_treedepth = 15))


summary(kf1)

Family: gaussian
  Links: mu = identity; sigma = identity
Formula: kn_flex_cc_Nm ~ s(angle_ext_ref0) + (angle_ext_ref0 | id)
   Data: kf (Number of observations: 844)
Samples: 2 chains, each with iter = 2000; warmup = 1000; thin = 1;
         total post-warmup samples = 2000

Smooth Terms:
                       Estimate Est.Error l-95% CI u-95% CI Eff.Sample Rhat
sds(sangle_ext_ref0_1)   114.17     28.09    72.93   181.78        634 1.01

Group-Level Effects:
~id (Number of levels: 11)
                              Estimate Est.Error l-95% CI u-95% CI Eff.Sample Rhat
sd(Intercept)                    25.61      6.48    16.39    40.52        967 1.00
sd(angle_ext_ref0)                0.44      0.12     0.28     0.72        636 1.00
cor(Intercept,angle_ext_ref0)    -0.30      0.27    -0.74     0.28        913 1.00

Population-Level Effects:
                  Estimate Est.Error l-95% CI u-95% CI Eff.Sample Rhat
Intercept           102.20      8.23    85.83   118.71        596 1.01
sangle_ext_ref0_1   -52.89      7.70   -67.51   -37.92       2000 1.00

Family Specific Parameters:
      Estimate Est.Error l-95% CI u-95% CI Eff.Sample Rhat
sigma     6.79      0.17     6.47     7.13       2000 1.00

Samples were drawn using sampling(NUTS). For each parameter, Eff.Sample
is a crude measure of effective sample size, and Rhat is the potential
scale reduction factor on split chains (at convergence, Rhat = 1).
```

```
kf2<-brm(bf(kn_flex_ecc_Nm ~ s(angle_ext_ref0) + (angle_ext_ref0|id)),
      data = kf,
      family = gaussian(),
      chains = 4,
      control = list(adapt_delta = 0.95,max_treedepth = 15))


summary(kf2)

Family: gaussian
  Links: mu = identity; sigma = identity
Formula: kn_flex_ecc_Nm ~ s(angle_ext_ref0) + (angle_ext_ref0 | id)
   Data: kf (Number of observations: 844)
Samples: 2 chains, each with iter = 2000; warmup = 1000; thin = 1;
         total post-warmup samples = 2000

Smooth Terms:
                       Estimate Est.Error l-95% CI u-95% CI Eff.Sample Rhat
sds(sangle_ext_ref0_1)   136.76     39.02    78.85   230.52        881 1.00

Group-Level Effects:
~id (Number of levels: 11)
                              Estimate Est.Error l-95% CI u-95% CI Eff.Sample Rhat
sd(Intercept)                    85.89     19.19    57.10   134.61        695 1.01
sd(angle_ext_ref0)                1.16      0.27     0.77     1.81        683 1.01
cor(Intercept,angle_ext_ref0)    -0.80      0.13    -0.95    -0.45        830 1.01

Population-Level Effects:
                  Estimate Est.Error l-95% CI u-95% CI Eff.Sample Rhat
Intercept           198.38     16.26   165.50   231.46        621 1.00
sangle_ext_ref0_1    68.10     21.78    26.49   112.56       1155 1.00

Family Specific Parameters:
      Estimate Est.Error l-95% CI u-95% CI Eff.Sample Rhat
sigma    24.49      0.59    23.40    25.61       2000 1.00

Samples were drawn using sampling(NUTS). For each parameter, Eff.Sample
is a crude measure of effective sample size, and Rhat is the potential
scale reduction factor on split chains (at convergence, Rhat = 1).
```

```
#hamstrings by quadriceps at each angular position as dependent variable"

kf3<-brm(bf(H_Q~ s(angle_ext_ref0) + (1|id)),

      data = kf, family = gaussian(),

      chains = 4, control = list(adapt_delta = 0.99,max_treedepth = 15))


summary(kf3)

Family: gaussian
  Links: mu = identity; sigma = identity
Formula: H_Q ~ s(angle_ext_ref0) + (1 | id)
   Data: kf (Number of observations: 844)
Samples: 2 chains, each with iter = 2000; warmup = 1000; thin = 1;
         total post-warmup samples = 2000

Smooth Terms:
                       Estimate Est.Error l-95% CI u-95% CI Eff.Sample Rhat
sds(sangle_ext_ref0_1)     0.41      0.27     0.12     1.16        414 1.01

Group-Level Effects:
~id (Number of levels: 11)
              Estimate Est.Error l-95% CI u-95% CI Eff.Sample Rhat
sd(Intercept)     0.21      0.05     0.13     0.33        340 1.00

Population-Level Effects:
                  Estimate Est.Error l-95% CI u-95% CI Eff.Sample Rhat
Intercept             0.58      0.07     0.44     0.72        295 1.00
sangle_ext_ref0_1    -0.06      0.11    -0.35     0.11        499 1.00

Family Specific Parameters:
      Estimate Est.Error l-95% CI u-95% CI Eff.Sample Rhat
sigma     0.17      0.00     0.16     0.18       2000 1.00

Samples were drawn using sampling(NUTS). For each parameter, Eff.Sample
is a crude measure of effective sample size, and Rhat is the potential
scale reduction factor on split chains (at convergence, Rhat = 1).
```

```
#Figures codes


plot.ke1<-marginal_effects(ke1)

plot(plot.ke1)[[1]] +xlab("Knee angular position, degrees")+ylab("Concentric knee extension moment of force, N.m")+scale_x_continuous(breaks=c(0,10,20,30,40,50,60,70,80,90))+scale_y_continuous(breaks=c(50,100,150,200,250,300))


plot.ke2<-marginal_effects(ke2)

plot(plot.ke2)[[1]] +xlab("Knee angular position, degrees")+ylab("Eccentric knee extension moment of force, N.m")+scale_x_continuous(breaks=c(0,10,20,30,40,50,60,70,80,90))+scale_y_continuous(breaks=c(50,100,150,200,250,300))


plot.ke3<-marginal_effects(ke3)

plot(plot.ke3)[[1]] +xlab("Knee angular position, degrees")+ylab("Functional hamstring by quadriceps ratio for knee extension")+scale_x_continuous(breaks=c(0,10,20,30,40,50,60,70,80,90))+scale_y_continuous(breaks=c(0.5,1.0,1.5,2.0,2.5,3.0))


plot.kf1<-marginal_effects(kf1)

plot(plot.kf1)[[1]] +xlab("Knee angular position, degrees")+ylab("Concentric knee flexion moment of force, N.m")+scale_x_continuous(breaks=c(0,10,20,30,40,50,60,70,80,90))+scale_y_continuous(breaks=c(50,75,100,125,150,175))



plot.kf2<-marginal_effects(kf2)

plot(plot.kf2)[[1]] +xlab("Knee angular position, degrees")+ylab("Eccentric knee flexion moment of force, N.m")+scale_x_continuous(breaks=c(0,10,20,30,40,50,60,70,80,90))+scale_y_continuous(breaks=c(50,100,150,200,250,300))


plot.kf3<-marginal_effects(kf3)

plot(plot.kf3)[[1]] +xlab("Knee angular position, degrees")+ylab("Functional hamstring by quadriceps ratio for knee flexion")+scale_x_continuous(breaks=c(0,10,20,30,40,50,60,70,80,90))+scale_y_continuous(breaks=c(0.2,0.3,0.4,0.5,0.6,0.7,0.8,0.9,1.0))
```
